# ﻿A braconid wasp (Hymenoptera, Braconidae) from the Lower Cretaceous amber of San Just, eastern Iberian Peninsula

**DOI:** 10.3897/zookeys.1103.83650

**Published:** 2022-05-30

**Authors:** Sergio Álvarez-Parra, Enrique Peñalver, Xavier Delclòs, Michael S. Engel

**Affiliations:** 1 Departament de Dinàmica de la Terra i de l’Oceà and Institut de Recerca de la Biodiversitat (IRBio), Facultat de Ciències de la Terra, Universitat de Barcelona, c/ Martí i Franquès s/n, 08028, Barcelona, Spain; 2 Instituto Geológico y Minero de España-CSIC, c/ Cirilo Amorós 42, 46004, Valencia, Spain; 3 Division of Entomology, Natural History Museum, University of Kansas, 1501 Crestline Drive – Suite 140, Lawrence, Kansas 66045-4415, USA; 4 Department of Ecology and Evolutionary Biology, University of Kansas, Lawrence, Kansas 66045, USA; 5 Division of Invertebrate Zoology, American Museum of Natural History, Central Park West at 79; 6 th; 7 Street, New York, New York 10024-5192, USA

**Keywords:** Albian, fossil, Ichneumonoidea, Protorhyssalinae, Spanish amber, taxonomy, wasp diversity, wing venation

## Abstract

Braconid parasitoid wasps are a widely diversified group today, while their fossil record from the Mesozoic is currently poorly known. Here, we describe *Utrillabraconelectropteron* Álvarez-Parra & Engel, **gen. et sp. nov.**, from the upper Albian (Lower Cretaceous) amber of San Just in the eastern Iberian Peninsula. The holotype specimen is incomplete, although the forewing and hind wing venation are well preserved. The new taxon is assigned to the subfamily †Protorhyssalinae (Braconidae) and, based on characteristics of the wing venation, seems to be closely related to *Protorhyssalusgoldmani* Basibuyuk & Quicke, 1999 and *Diorhyssalusallani* (Brues, 1937), both from Upper Cretaceous ambers of North America. We discuss the taxonomy of the Cretaceous braconids, considering †Seneciobraconinae as a valid subfamily. We also comment on possible relationships within †Protorhyssalinae, although a phylogenetic analysis is necessary. Additionally, a checklist is included of braconids known from Cretaceous ambers.

## ﻿Introduction

Braconidae are the second largest family of Hymenoptera in terms of species numbers (Chen and van Achterberg 2019), trailing just behind the closely related family, Ichneumonidae. Like ichneumonids, braconids are parasitoid wasps, with their larvae developing within or externally on other insects, typically Coleoptera, Diptera, and Lepidoptera, but actually encompassing a considerable breadth of hosts from aphids to other wasps, and even adult stages (e.g., Euphorinae) (Wharton 1993). Given that braconids attack the immatures of many agriculturally important pest species, they have been heavily employed in sustainable pest management programs throughout the world (e.g., Nomano et al. 2015).

Braconids belong to the superfamily Ichneumonoidea, which comprises the extant families Ichneumonidae, Braconidae, and Trachypetidae (Quicke et al. 2020), along with the extinct †Praeichneumonidae, a monogeneric family including five species known from Early Cretaceous compression fossils (Rasnitsyn 1983, 1990; Kopylov 2012). A putative fifth group, †Ichneumonomimidae (Rasnitsyn 1975), has subsequently been considered to belong to Trigonalyidae (Rasnitsyn 1988), while the Trachypetidae has been recently restored as a non-cyclostome braconid subfamily (Jasso-Martínez et al. 2022a, 2022b). The fossil record of Ichneumonoidea is most diverse in Cenozoic deposits but extends well into the Early Cretaceous, with Mesozoic fossils representing early diverging lineages of both Ichneumonidae and Braconidae, several of which have been difficult to place phylogenetically or to even confirm as monophyletic (Kopylov et al. 2021; Spasojevic et al. 2021; Viertler et al. 2022).

**Figure 1.**
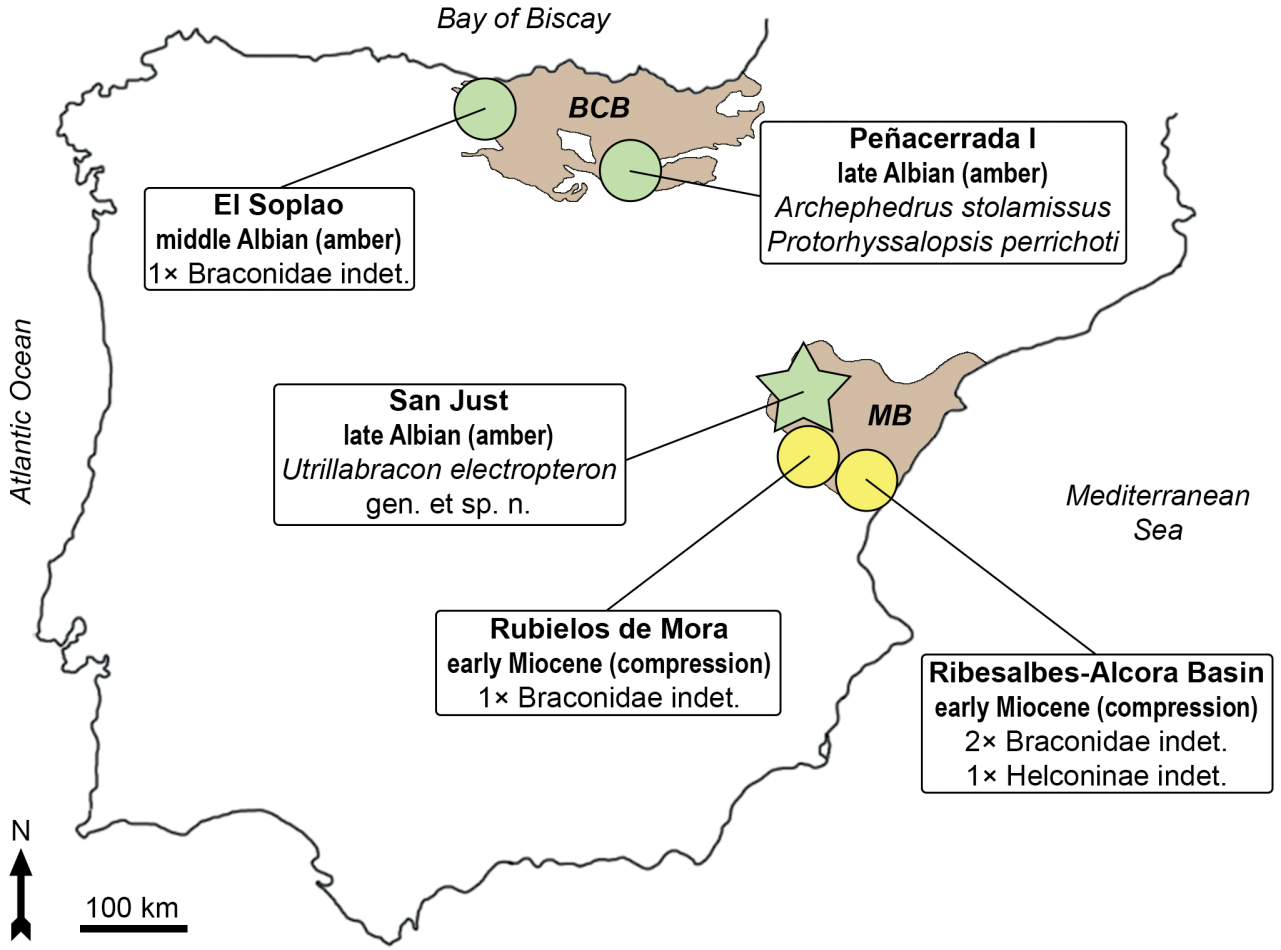
Map of the Iberian Peninsula showing the location of the amber and compression outcrops that have yielded braconid wasps. Basque-Cantabrian (BCB) and Maestrazgo (MB) basins are represented. The type locality the studied specimen is indicated with a star. The specimens from El Soplao and Rubielos de Mora are undescribed to date.

One notable example of these early lineages is the braconid subfamily †Protorhyssalinae, a group of parasitoid wasps almost exclusively known by amber inclusions from the Albian to the Campanian (Li et al. 2021). Braconidae are currently represented by 21 genera and 22 species in Cretaceous ambers (Table 1), besides other specimens preserved as compressions in Cretaceous rocks (Belokobylskij 2012). Only two braconid species have been previously reported from Cretaceous Spanish amber (Ortega-Blanco et al. 2009, 2011) (Fig. 1). Furthermore, other specimens of the family were found in lower Miocene compression outcrops from the eastern Iberian Peninsula (Peñalver and Martínez-Delclòs 2000; Álvarez-Parra and Peñalver 2019). Here, we describe a new genus and species of fossil wasp belonging to the subfamily †Protorhyssalinae included in amber from the upper Albian San Just in the eastern Iberian Peninsula. Although the specimen is incomplete, the wings are extraordinarily well preserved and allow for its proper placement and characterization relative to other protorhyssalines. We provide a description of the new species and compare it with the previously known genera of †Protorhyssalinae. In addition, we append comments on the diversity of the subfamily and putative phylogenetic groups among this assemblage of wasps.

**Table 1.** Checklist of species of Braconidae (Hymenoptera, Ichneumonoidea) from Cretaceous ambers. The two species marked with an asterisk need taxonomic revision. For Cretaceous compression fossils see Belokobylskij (2012).

Subfamily	Genus and species	Locality	Age	Reference
Aphidiinae	*Archephedrusstolamissus* Ortega-Blanco, Bennett, Delclòs, & Engel, 2009	Peñacerrada I, Spain	late Albian	Ortega-Blanco et al. (2009)
Brachistinae	“*Neoblacus*” (=*Blacus*) *facialis* Brues, 1937 *	Cedar Lake, Canada	Campanian	Brues (1937)
Euphorinae	“*Pygostolus*” *patriarchicus* Brues, 1937 *	Cedar Lake, Canada	Campanian	Brues (1937)
†Megalyrhyssalinae	*Megalyrhyssalusclavicornis* Belokobylskij & Jouault, 2021	Hukawng Valley, Myanmar	early Cenomanian	Belokobylskij and Jouault (2021)
†Protobraconinae	*Rhetinorhyssalitesemersoni* Engel, Thomas, & Alqarni, 2017	Sayreville, USA	Turonian	Engel et al. (2017); Chen et al. (2021b)
	*Chainochorasyntoma* Chen & van Achterberg, 2021	Hukawng Valley, Myanmar	early Cenomanian	Chen et al. (2021a)
	*Kleistochoradolichura* Chen & van Achterberg, 2021	Hukawng Valley, Myanmar	early Cenomanian	Chen et al. (2021a)
	*Protobraconrobusticauda* Chen & van Achterberg, 2021	Hukawng Valley, Myanmar	early Cenomanian	Chen et al. (2021b)
	*Tibialobraconcompressicornis* Chen & van Achterberg, 2021	Hukawng Valley, Myanmar	early Cenomanian	Chen et al. (2021b)
†Protorhyssalinae	*Diorhyssalusallani* (Brues, 1937)	Cedar Lake, Canada	Campanian	Brues, (1937); Engel (2016); Chen et al. (2021b)
	*Protorhyssalusgoldmani* Basibuyuk & Quicke, 1999	Sayreville, USA	Turonian	Basibuyuk et al. (1999)
	*Protorhyssalodesarnaudi* Perrichot, Nel, & Quicke, 2009	Cadeuil, France	early Cenomanian	Perrichot et al. (2009); Chen et al. (2021b)
	*Archaeorhyssalussubsolanus* Engel, 2016	Hukawng Valley, Myanmar	early Cenomanian	Engel and Wang (2016)
	*Burmabracongracilens* Li, Shih, & Ren, 2021	Hukawng Valley, Myanmar	early Cenomanian	Li et al. (2021)
	*Burmabracongrossus* Li, Shih, & Ren, 2021	Hukawng Valley, Myanmar	early Cenomanian	Li et al. (2021)
	*Protorhyssalopsisperrichoti* Ortega-Blanco, Delclòs, & Engel, 2011	Peñacerrada I, Spain	late Albian	Ortega-Blanco et al. (2011)
	***Utrillabraconelectropteron* Álvarez-Parra & Engel, gen. et sp. n.**	San Just, Spain	late Albian	This paper
†Seneciobraconinae	*Seneciobraconnovalatus* Engel & Huang, 2018	Hukawng Valley, Myanmar	early Cenomanian	Engel et al. (2018)
*Incertae sedis*	*Aenigmabraconcapdoliensis* Perrichot, Nel, & Quicke, 2009	Cadeuil, France	early Cenomanian	Perrichot et al. (2009)
	*Pyramidibraconclypeatus* Chen & van Achterberg, 2021	Hukawng Valley, Myanmar	early Cenomanian	Chen et al. (2021b)
	*Rhetinorhyssalusmorticinus* Engel, 2016	Hukawng Valley, Myanmar	early Cenomanian	Engel (2016)
	*Stephanorhyssaluslongiscapus* Belokobylskij & Jouault, 2021	Hukawng Valley, Myanmar	early Cenomanian	Belokobylskij and Jouault (2021)

## ﻿Materials and methods

The amber material reported here comes from the San Just amber-bearing outcrop (Teruel Province, Aragón, Spain). The site is located near the Utrillas Municipality, in the Aliaga Sub-basin within the Maestrazgo Basin (Fig. 1). More than 30 amber-bearing outcrops have been reported in this basin, although only four of them have yielded bioinclusions (Álvarez-Parra et al. 2021). Stratigraphically, the San Just section has been assigned to the Escucha Formation (Peñalver et al. 2007). The amber-rich level is composed of grey-black marls with a high content of organic matter, charcoal, and fusinite and has been interpreted as a freshwater swamp plain (Peñalver et al. 2007; Villanueva-Amadoz et al. 2010). The site was dated as middle–earliest upper Albian based on palynological evidence (Villanueva-Amadoz et al. 2010). A new palynological study constrains the dating to the upper Albian (Eduardo Barrón pers. comm.). San Just is the type locality of 26 arthropod species (including the new species here described) and the Hymenoptera are represented by nine species in eight families (Santer et al. 2022). The amber piece was recovered during an excavation in 2012 (Government of Aragón permit 119/10-11-2012). The original amber piece was divided in four epoxy preparations to better examine the syninclusions. This process followed the methodology of Corral et al. (1999). The specimen was photographed and drawn using an Olympus CX41 compound microscope, with an attached digital camera sCMEX-20 and a camera lucida. Photographs were made using the software ImageFocusAlpha v. 1.3.7.12967.20180920 and the figures were prepared using Photoshop CS6. Venational nomenclature is based on Huber and Sharkey (1993) and Ortega-Blanco et al. (2009). The specimen is deposited in the Museo Aragonés de Paleontología (Fundación Conjunto Paleontológico de Teruel-Dinópolis), Teruel, Spain. The fossil notation “MAP” corresponds to the number at the Museo Aragonés de Paleontología, while “SJE2012” is the field number.

### ﻿Systematic paleontology

#### Family Braconidae Nees von Esenbeck, 1811

##### ﻿Subfamily † Protorhyssalinae

Taxon classificationAnimaliaHymenopteraBraconidae

Basibuyuk, Quicke, & van Achterberg, 1999

5985C010-EAB7-519B-89C4-D39F47E88909

Protorhyssalinae Basibuyuk, Quicke, & van Achterberg, 1999: 211. Type genus: Protorhyssalus Basibuyuk & Quicke in Basibuyuk et al. (1999), by original designation.

###### Comments.

Herein we restore the traditional concept of †Protorhyssalinae as recognized by Basibuyuk et al. (1999) and Chen and van Achterberg (2019). Belokobylskij and Jouault (2021) proposed a classification in which virtually all Cretaceous braconids are thrown into a paraphyletic group, rendering †Protorhyssalinae a meaningless grade. Admittedly, restoring †Protorhyssalinae still leaves the group paraphyletic but at least removes the more obviously derived groups and thereby narrows the challenge as to the affinities of the remaining genera. Nonetheless, while Belokobylskij and Jouault (2021) advocated for such a paraphyletic assemblage, they used plesiomorphic features along with autapomorphies to establish the subfamily †Megalyrhyssalinae. Unfortunately, †Megalyrhyssalinae is poorly justified and could be merely an autapomorphic form of the same protorhyssaline grade. By their own reasoning, they should have either not established such a subfamily or further divided †Protorhyssalinae to resolve the paraphyly. Under their conception of †Protorhyssalinae, †Megalyrhyssalinae would be a junior synonym. For now, we recognize the following subfamilies: †Protorhyssalinae, †Seneciobraconinae (*Seneciobracon*), and †Megalyrhyssalinae (*Megalyrhyssalus*), noting that the last may not be sufficiently justified but may well be worth considering once the full phylogeny of the genera comprising these groups is elucidated. Until such time it seems that further alterations of the subfamilial system in the absence of a cladistic framework would be unwarranted.

###### Included genera and species.

*Archaeorhyssalussubsolanus* Engel, 2016; *Burmabracongracilens* Li, Shih, & Ren, 2021; *B.grossus* Li, Shih, & Ren, 2021; *Diorhyssalusallani* (Brues, 1937); *Protorhyssalodesarnaudi* Perrichot, Nel, & Quicke, 2009; *Protorhyssalopsisperrichoti* Ortega-Blanco, Delclòs, & Engel, 2011; *Protorhyssalusgoldmani* Basibuyuk & Quicke, 1999; and *Utrillabraconelectropteron* Álvarez-Parra & Engel, gen. et sp. nov. *Cretorhyssalusbrevis* Belokobylskij, 2012, *Magadanobraconrasnitsyni* Belokobylskij, 2012, and *M.zherikhini* Belokobylskij, 2012, known from compression fossils, were putatively assigned to †Protorhyssalinae*sensu*Belokobylskij (2012).

##### ﻿ Utrillabracon

Taxon classificationAnimaliaHymenopteraBraconidae

Álvarez-Parra & Engel gen. nov.

1A91A0D8-79D6-5C51-8C94-6A31321FADF0

http://zoobank.org/C6FE19C1-A5D0-4780-9860-F611198EF09C

###### Type species.

*Utrillabraconelectropteron* Álvarez-Parra & Engel, sp. nov.

###### Diagnosis.

Forewing with margin bearing setae; pterostigma 4 × longer than wide; 1Rs relatively long and curved; r-rs oblique, arising medially from pterostigma; r-rs several times longer than abscissa of M between 2Rs and m-cu; marginal cell reaching wing apex; rs-m nebulous; elongate, five-sided second submarginal cell, 3 × longer than wide; 1M and m-cu of similar length; m-cu distinctly postfurcal; 2m-cu absent; cu-a slightly postfurcal and orthogonal. Hind wing with margin bearing setae; R1 distally widened with several hamuli beyond its apex; Sc + R not aligned with Rs; 2Cu present. Pretarsal claws present, without preapical tooth; arolium wide.

###### Etymology.

The generic name is a combination of Utrillas, municipality where the San Just amber outcrop is located, and *Bracon* Fabricius, 1804, type genus of the family Braconidae. The gender of the name is masculine.

##### ﻿ Utrillabracon electropteron

Taxon classificationAnimaliaHymenopteraBraconidae

Álvarez-Parra & Engel sp. nov.

428A8EF6-293C-5643-A08E-8CD20C399063

http://zoobank.org/59B73E2C-0514-4DA4-8A87-ABF61D6EF2A8

Fig. 2

###### Material.

***Holotype***, MAP-7819 (SJE2012 49-04), sex unknown, from San Just amber. The holotype is largely preserved as the forewings and hind wings. Some parts of the head, an antenna, and a leg are next to the wings. Undetermined cuticular fragments are visible near the wings. Deposited in the Museo Aragonés de Paleontología (Fundación Conjunto Paleontológico de Teruel-Dinópolis) in Teruel, Spain. Syninclusions include three other hymenopterans (probable serphitid, platygastrid, and stigmaphronid wasps). The holotype is prepared isolated in an epoxy prism of 20 × 15 mm.

**Figure 2.**
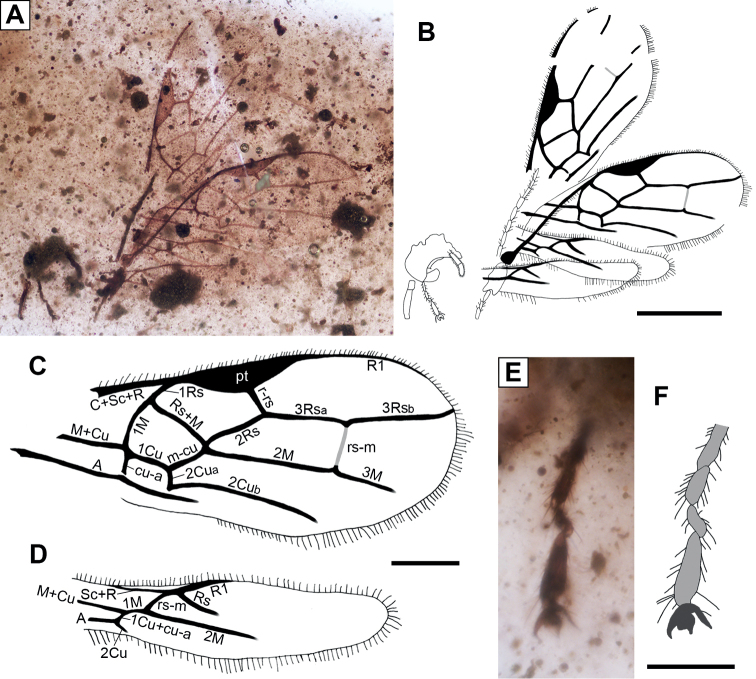
*Utrillabraconelectropteron* Álvarez-Parra & Engel, gen. et sp. nov. (Braconidae, †Protorhyssalinae) from the upper Albian amber-bearing outcrop of San Just, specimen MAP-7819 (SJE2012 49-04). **A, B** photograph and drawing of preserved remains, both to the same scale **C** forewing venation **D** hind wing venation **E, F** photograph and drawing of tarsus and pretarsus, both to the same scale. Abbreviation: pt = pterostigma. Scale bars: 0.5 mm (**A, B**); 0.2 mm (**C, D**); 0.1 mm (**E, F**).

###### Locality and horizon.

San Just amber-bearing outcrop, Utrillas, Teruel, Spain; Maestrazgo Basin, Escucha Formation, upper Albian (Peñalver et al. 2007).

###### Diagnosis.

As for the genus (*vide supra*).

###### Description.

Head deformed and incomplete as preserved (Fig. 2A, B); antenna partially preserved with 11 flagellomeres covered by setae, multiporous plate sensilla not visible; only distal two maxillary palpomeres preserved, covered by fine setae. Forewings and venation rather complete (Fig. 2C), forewing base not preserved, more than 1.31 mm long and 0.53 mm in its maximum width, margin bearing setae; C + Sc + R fused anterobasally, extending along wing margin to pterostigma; pterostigma 4 × longer than wide (0.33 mm vs 0.08 mm); elongate marginal cell, 3 × longer than wide (0.57 mm vs 0.19 mm), reaching wing apex; 1Rs relatively long and curved; Rs + M slightly sinuous; first submarginal cell 2 × longer than wide (0.31 mm vs 0.15 mm), pentagonal; 2Rs slightly sinuous; r-rs oblique, arising medially from pterostigma, 0.08 mm long; 3Rs extending nearly straight until wing margin, 0.55 mm long; r-rs several times longer than abscissa of M between 2Rs and m-cu; 1M curved, 2 × longer than 1Rs (0.14 mm vs 0.07 mm); 2M straight, 0.38 mm long; almost straight 3M, disappearing before wing margin; rs-m nebulous, 0.13 mm long; elongate, pentagonal second submarginal cell, 3 × longer than wide (0.38 mm vs 0.13 mm); trapezoidal third submarginal cell, 0.31 mm long; first discal cell almost 2 × longer than wide (0.21 mm vs 0.12 mm); m-cu distinctly postfurcal (absence of a vein 2Rs + M), 0.12 mm long; lacking 2m-cu; elongate second discal cell, 0.63 mm long; cu-a (nervulus) slightly postfurcal (therefore presence of an exceptionally short 1Cu_a_), 0.06 mm long, perpendicular to 1Cu and A; 1Cu nearly straight, 0.14 mm long; 2Cu strongly curved basally separating 2Cu_a_ (0.05 mm long) and 2Cu_b_, latter curved and directed towards wing margin (but without meeting margin); first subdiscal cell 2 × longer than wide (0.13 mm vs 0.07 mm); elongate and narrow second subdiscal cell; A tubular and nearly straight; 1a and 2a not visible. Hind wings and venation rather complete (Fig. 2D), hind wing base not preserved, more than 0.94 mm long and 0.23 mm at its maximum width, margin bearing setae; Sc + R fused anterobasally; R1 distally widened with several hamuli beyond its apex; Sc + R not aligned with Rs; 1M short, 0.05 long; rs-m oblique, 0.07 mm long; Rs and M ending as nebulous veins before margin; 1Cu + cu-a inclivitous, 0.03 mm long; short 2Cu, not contacting wing margin. Two fragments of legs visible: a partial femur and a tarsus; four distal tarsomeres preserved covered by fine setae (Fig. 2E, F), tarsomere III 0.06 mm long, tarsomere IV 0.04 mm long, tarsomere V 0.08 mm long; pretarsus with paired claws, preapical tooth absent, arolium wide.

###### Etymology.

The specific epithet is a combination of the Greek *ἤλεκτρον* (élektron), meaning, “amber”, and *πτηνόν* (ptéron), meaning, “winged creature”, and referring to the fact that the holotype is mainly preserved by the wings in amber.

## ﻿Discussion

The newly reported San Just amber wasp can be assigned to Braconidae quite easily owing to the characteristic wing venation: Rs + M present and 2m-cu absent in the forewing and rs-m proximal to bifurcation of R1 and Rs in the hind wing (Huber and Sharkey 1993; Belokobylskij and Jouault 2021). The absence of 2m-cu in the forewing also serves to exclude the fossil from the plesiomorphic †Praeichneumonidae. Additionally, the Trachypetinae (formerly as family Trachypetidae) have rs-m distal to the separation of R1 and Rs (Quicke et al. 2020), and therefore the current fossil also does not accord with the circumscription of this group. Although many have noted that braconid wing venation can be quite variable, the current fossil from San Just cannot be ascribed to any other clade and is quite readily attributable to Braconidae. In fact, several Cretaceous braconids possess 2m-cu in the forewing, such as *Aenigmabraconcapdoliensis* Perrichot, Nel, & Quicke, 2009 (subfamily *incertae sedis*), *Stephanorhyssaluslongiscapus* Belokobylskij & Jouault, 2021 (subfamily *incertae sedis*), and species of the subfamily †Eoichneumoninae, all of which likely retain this trait symplesiomorphically (Belokobylskij and Jouault 2021). Furthermore, some living species of the subfamilies Apozyginae, Doryctinae, and Rhyssalinae (all of crown-Braconidae) possess 2m-cu in the forewing (Tobias and Belokobylskij 1983), while some species of a few subfamilies of Ichneumonidae lack this vein (Tobias 1963). All of these cases are easily identified as secondary reappearances of the crossvein or “atavisms” based on the phylogenetic placement of the taxa in question (Belokobylskij and Jouault 2021).

The presence of a pentagonal (five-sided) second submarginal cell in the forewing and vein 2Cu in the hind wing indicates that *Utrillabraconelectropteron* is currently best assigned to the subfamily †Protorhyssalinae (Basibuyuk et al. 1999; Chen et al. 2021b), despite the fact that this group, even in its restricted sense, may be paraphyletic. Indeed, the overall venation of *Utrillabracon* accords broadly with that of †Protorhyssalinae (Basibuyuk et al. 1999). The pentagonal second submarginal cell in the forewings is likely to be plesiomorphic in braconids. The other braconid subfamilies with a Cretaceous record, such as Aphidiinae, †Seneciobraconinae, †Megalyrhyssalinae, and †Protobraconinae, lack 2Cu in the hind wing (Belokobylskij and Jouault 2021; Chen et al. 2021b). Several extant braconid subfamilies have 2Cu in the hind wing (Perrichot et al. 2009; Belokobylskij and Jouault 2021), and interestingly they are phylogenetically placed basal to all other crown-braconids (Apozyginae) or to the derived non-cyclostome lineage (Acampsohelconinae, Agathidinae, Meteorideinae, and Sigalphinae) (Chen and van Achterberg 2019). Furthermore, this character is also present in some †Eoichneumoninae (Braconidae), and in the ichneumonoid groups Trachypetinae (Braconidae), †Praeichneumonidae, and Ichneumonidae (Belokobylskij and Jouault 2021). Therefore, it is probable that the presence of 2Cu in the hind wing is symplesiomorphic across all of these lineages (Perrichot et al. 2009; Belokobylskij and Jouault 2021). The †Eoichneumoninae possess 2m-cu in the forewings (like the †Praeichneumonidae and the vast majority of Ichneumonidae) (Belokobylskij and Jouault 2021; Chen et al. 2021b), and quite unlike *U.electropteron*.

The San Just fossil may be easily distinguished from the two unplaced Canadian Late Cretaceous amber species “*Neoblacus*” (= *Blacus*) *facialis* Brues, 1937 and “*Pygostolus*” *patriarchicus* Brues, 1937. Both of these species need revision and likely do not belong to the genera to which Brues assigned them (Antropov et al. 2014; Chen et al. 2021b). Nonetheless, both are sufficiently known as to differentiate them from *U.electropteron*. The species *N.* (= *B.*) *facialis* lacks Rs + M and rs-m in the forewing (vs present), r-rs arises before the middle of the pterostigma and is perpendicular to the costal margin (vs inclivitous and arising pterostigmal midlength), and cu-a is distinctly postfurcal (vs slightly postfurcal) (Brues 1937). The pterostigma of *U.electropteron* seems to be similar to that of *N.* (= *B.*) *facialis*, as in both species it is 4 × longer than wide (Brues 1937). “*Pygostolus*” *patriarchicus* has a triangular pterostigma with basal and apical margins of equal length (vs pterostigma long and narrow), and cu-a postfurcal in the forewing (Brues 1937). The *incertae sedis* braconids *A.capdoliensis* and *S.longiscapus* differ from *U.electropteron* in the presence of 2m-cu and cu-a postfurcal in the forewing (Perrichot et al. 2009; Belokobylskij and Jouault 2021). *Pyramidibraconclypeatus* Chen & van Achterberg, 2021 and *Rhetinorhyssalusmorticinus* Engel, 2016 are currently not assigned to a subfamily and differ from *U.electropteron* in several characters, such as cu-a strongly inclivitous in the forewing, Sc + R aligned with Rs, and both lack 2Cu in the hind wing (Engel 2016; Chen et al. 2021b).

Considering those genera currently assigned to †Protorhyssalinae, *U.electropteron* can be differentiated from them as summarized below. *Archaeorhyssalussubsolanus* lacks 1Rs (vs present), has a distinct 2Rs + M (vs absent), and m-cu antefurcal and contacting Rs + M (vs not contacting) in the forewing (Engel and Wang 2016). *Burmabracongracilens*, *B.grossus*, and *Protorhyssalopsisperrichoti* have Sc + R aligned with Rs in the hind wing (vs not aligned), aside from a slew of further differences (Li et al. 2021; Ortega-Blanco et al. 2011). *Protorhyssalodesarnaudi* has cu-a distinctly postfurcal with 1Cu_a_ as long as cu-a (vs cu-a slightly postfurcal) in the forewing and also Sc + R aligned with Rs in the hind wing (Perrichot et al. 2009). The wing venation of *U.electropteron* is quite similar to that of *Protorhyssalusgoldmani* and *Diorhyssalusallani* (Brues 1937; Basibuyuk et al. 1999; Engel 2016). *Utrillabraconelectropteron* shares with *P.goldmani* the marginal cell reaching the wing apex, vein m-cu postfurcal, and cu-a slightly postfurcal in the forewing, while differing in the length of the second submarginal cell (shorter in *P.goldmani*) and the length of r-rs in comparison to the abscissa of M between 2Rs and m-cu (similar length in *P.goldmani* and several times longer in *U.electropteron*) (Basibuyuk et al. 1999). Both species have Sc + R not aligned with Rs in the hind wing (Basibuyuk et al. 1999). In general, the venation of *U.electropteron* seems to be closest to that of *D.allani* (Brues 1937; Engel 2016). Particularly, the lengths of the second submarginal cell and r-rs (several times longer than the abscissa of M between 2Rs and m-cu) are similar in both, and they also have m-cu postfurcal (Brues 1937; Engel 2016). The characters present in *U.electropteron* that differ from *D.allani* are 1Rs curved (vs shorter and straight), rs-m nebulous (vs sclerotized), and cu-a orthogonal and slightly postfurcal (vs inclivitous and somewhat more postfurcal) (Brues 1937; Engel 2016). The hind wing of *D.allani* is poorly known (Engel 2016). Therefore, despite the similar venation of the San Just species with *D.allani*, we prefer to assign it to a new genus, as we think that the anatomical differences cannot be associated with variability between species. Furthermore, the San Just species and *D.allani* are separated by more than 20 Myr (Albian to Campanian), and a vast geographical distance (Iberian Peninsula vs western Canada).

Based on the similarities of the wing venations of *U.electropteron*, *P.goldmani*, and *D.allani*, it is possible that they were closely related. These three taxa may form a group within †Protorhyssalinae, supported by the following characters: 1Rs present, pterostigma long and narrow, r-rs arising medially from pterostigma, m-cu distinctly postfurcal, cu-a slightly postfurcal (1Cu_a_ shorter than cu-a) in the forewing, and Sc + R not aligned with Rs in the hind wing. The latter character is tenuous for *D.allani*, as the hind wings are poorly documented (Brues 1937; Engel 2016). Nonetheless, it is probable that the hind wing of *D.allani* also had 2Cu, based on the other anatomical similarities with *P.goldmani* and *U.electropteron*. A revision of the holotype of *D.allani* or the discovery of new specimens of the same morphotype may demonstrate the presence of 2Cu (and Sc + R not aligned with Rs) for the hind wing, thus corroborating its placement to †Protorhyssalinae. *Archaeorhyssalussubsolanus* has m-cu antefurcal, a distinctive character among protorhyssalines, and it may be that this genus belongs to a more derived clade between the generally plesiomorphic †Protorhyssalinae and the more derived †Seneciobraconinae. We refrain, however, from establishing another monogeneric subfamily for this genus until such time as more critical cladistic work has been undertaken. *Burmabracongracilens*, *B.grossus*, *P.arnaudi*, and *P.perrichoti* share Sc + R aligned with Rs in the hind wing, a character that could be a potential apomorphy of a group formed by these four species. In any case, these groupings are based solely on observations of wing venation and a phylogenetic analysis incorporating larger suites of data is necessary to resolve monophyly (or lack thereof) for †Protorhyssalinae, relationships among the constituent groups, as well as the placement of the various extinct subfamilies among early diverging Braconidae. Basibuyuk et al. (1999) noted that the subfamily †Protorhyssalinae lacks apomorphies, and it is likely that it will be discovered to be a grade (Engel 2016; Chen and van Achterberg 2019), necessitating the removal of some genera to other or even new subfamilies (e.g., *Archaeorhyssalus*).

An interesting breadth of early braconid diversity is documented from Cretaceous amber inclusions and compression fossils (Table 1). Nonetheless, this diversity is trivial by comparison to the overwhelming diversity of present-day Braconidae (Chen and van Achterberg 2019). This may be the result of a Late Cretaceous diversification of the family, with little diversity present prior to this time. This may be partly the case as an incredible diversity of new potential hosts for braconids were appearing during the Late Cretaceous and into the Paleogene owing to the rise of several flower-associated insects at the time (Labandeira and Li 2021). However, there is likely also a considerable taphonomic bias against the capture and preservation of early fossil Braconidae (Martínez-Delclòs et al. 2004). Their typically diminutive size means that preservation in sediments requires exceptionally fine grains in order to have sufficient fidelity for their proper identification as braconids and despite the rich number of wasps included in amber, Cretaceous braconids are rare. This could be owing to the fact that braconids have little reason to be near resin flows except in the case of seeking or emerging from a host that was somehow present on or in trees exuding resins. Certainly, the family was present and widespread during the Cretaceous owing to their occurrence in deposits spanning Canada to Myanmar, and so the combination of potentially low abundances, lower than present species diversity, typically small body size necessitating exceptional preservational conditions, and biases away from resin-producing sources may account for their rarity. If this is the case, then it would also render challenging any direct exploration of their earliest history as fossils would likely continue to be rare.

## Supplementary Material

XML Treatment for Protorhyssalinae

XML Treatment for Utrillabracon

XML Treatment for Utrillabracon electropteron
